# A core-attachment based method to detect protein complexes in PPI networks

**DOI:** 10.1186/1471-2105-10-169

**Published:** 2009-06-02

**Authors:** Min Wu, Xiaoli Li, Chee-Keong Kwoh, See-Kiong Ng

**Affiliations:** 1 School of Computer Engineering, Nanyang Technological University, Singapore; 2 Institute for Infocomm Research, 1 Fusionopolis Way, Singapore

## Abstract

**Background:**

How to detect protein complexes is an important and challenging task in post genomic era. As the increasing amount of protein-protein interaction (PPI) data are available, we are able to identify protein complexes from PPI networks. However, most of current studies detect protein complexes based solely on the observation that dense regions in PPI networks may correspond to protein complexes, but fail to consider the inherent organization within protein complexes.

**Results:**

To provide insights into the organization of protein complexes, this paper presents a novel core-attachment based method (COACH) which detects protein complexes in two stages. It first detects protein-complex cores as the "hearts" of protein complexes and then includes attachments into these cores to form biologically meaningful structures. We evaluate and analyze our predicted protein complexes from two aspects. First, we perform a comprehensive comparison between our proposed method and existing techniques by comparing the predicted complexes against benchmark complexes. Second, we also validate the core-attachment structures using various biological evidence and knowledge.

**Conclusion:**

Our proposed COACH method has been applied on two different yeast PPI networks and the experimental results show that COACH performs significantly better than the state-of-the-art techniques. In addition, the identified complexes with core-attachment structures are demonstrated to match very well with existing biological knowledge and thus provide more insights for future biological study.

## Background

With the completion of many genome-sequencing projects, the focus in the post-genomic era has turned to proteomics. One important task in proteomics is to detect protein complexes based on the PPI data generated by various experimental technologies, *e.g*., yeast-two-hybrid [1], affinity purification [2-4] and others.

Protein complexes are molecular aggregations of proteins assembled by multiple protein-protein interactions. Many proteins are functional only after they are assembled into a protein complex and interact with other proteins in this complex. Multiple-protein complexes are key molecular entities to perform cellular functions. For example, the complex 'RNA polymerase II' transcribes genetic information into messages for ribosomes to produce proteins and complex 'Proteasome core particle' is involved in the degradation of proteins, which is an essential process within the cell.

Pair-wise protein interactions can be modeled as a graph or network, where vertices are proteins and edges are protein-protein interactions (PPI). Such a network modeling provides a new perspective to understand the complicated biological systems [5]. Since proteins in the same complex are highly interactive with each other, protein complexes generally correspond to dense subgraphs in the PPI network [6,7] and many previous studies have been proposed based on this observation. Cliques (fully connected subgraphs) [7,8] can be directly predicted as protein complexes. Traditional graph clustering algorithms can also be applied to detect dense clusters as protein complexes [9-11]. Markov clustering method (MCL) [9] simulates random walks within graphs and thus partition the PPI network into many non-overlapping dense clusters. Graph cuts (*e.g*., minimum cut and normalized cut [10]) are also used for graph partition and thus for detecting protein complexes. King et al. [11] recently proposed a graph clustering algorithm to detect protein complexes, which applied a restricted neighborhood searching with a cost function. Some other methods detect dense subgraphs as protein complexes by conducting local neighborhood search [12-15]. Additional information are also more and more exploited to improve the predictions, for example, functional information used in some above studies [11,14] and data of protein binding interfaces used in [16].

In addition, several recent studies for detecting protein complexes rely solely on TAP data [8,17,18]. These techniques consist of two stages. First, they defined specific scoring methods based on the purification records and selected protein interactions with high scores (both direct and indirect interactions) to construct reliable PPI networks (*e.g*., "Socio-Affinity" score in [17]). Second, they applied some existing methods to detect dense clusters in the reliable PPI networks as protein complexes, e.g., MCL is used in [18] and clique-mining is used in [8].

**Figure 1:**
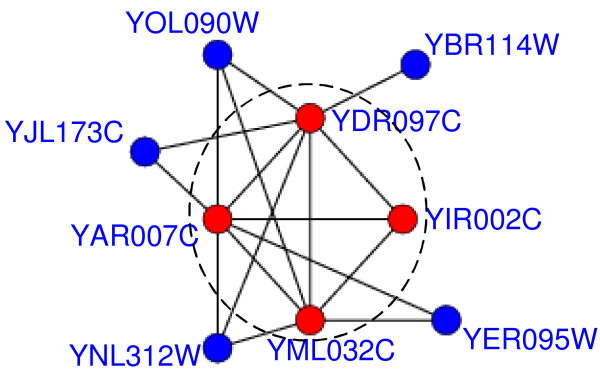
**DNA repair complex, which repairs DNA damage by interacting with damaged DNA**. Figure 1 shows an example of the DNA repair complex [17], whose core consists of four red proteins (YAR007C, YDR097C, YML032C and YIR002C) in the dashed circle. These four proteins form a fully connected subgraph (clique) in the PPI network. Proteins YBR114W, YER095W, YJL173C, YNL312W and YOL090W are the attachments of this complex. The interactions in this figure are from the DIP data.

Existing computational studies mainly focus on detecting highly connected subgraphs in PPI networks as protein complexes but ignore their inherent organization. However, recent analysis of experimentally detected protein complexes has revealed their inherent organization [19]. A protein complex generally contains a core in which proteins are highly co-expressed and share high functional similarity. The protein-complex core is often surrounded by some attachments, which assist the core to perform subordinate functions. Gavin et al.'s work [17] also demonstrates the similar architecture and modularity of protein complexes. Figure 1 shows an example of this kind of core-attachment architecture.

In this paper, we propose a new method, called COACH (**Co**re-**At**tac**h**ment based method), to detect protein complexes in PPI networks by considering their inherent organizations. In particular, protein-complex cores, as the "hearts" of the protein complexes, are first detected from each vertex's neighborhood graphs. We subsequently generate protein complexes by including attachments into the protein-complex cores. Experimental results using PPI data of *Saccharomyces cerevisiae *show that our COACH method does provide insights into the inherent modularity and organization of protein complexes. In addition, in terms of prediction accuracy, our COACH method also outperforms existing computational methods.

## Our proposed algorithm

A protein-protein interaction (PPI) network can be modeled as a simple graph *G *= (*V*, *E*), in which a vertex in vertex set *V *represents a protein and an edge in edge set *E *represents an interaction between two distinct proteins. This graph structure modeling is helpful for global analysis of PPI data, such as graph clustering for protein complex detection. Our COACH method operates in two phases. COACH first detects protein-complex cores and then applies an outward growing strategy to generate protein complexes by including attachments into the protein-complex cores. We will first briefly introduce some basic terminologies and then describe in detail our proposed method for protein complex detection.

### Preliminaries

Given a PPI network *G *= (*V*, *E*), the degree of a vertex *v *∈ *V *is the number of *v*'s neighbors in *G*, written as *deg*(*v*). The average degree of graph *G *is defined as the average of *deg*(*u*) for all *u *∈ *V*, written as *Avdeg*(*G*) in equation 1. The density of *G*, denoted as *den*(*G*), is defined in the equation 2.

(1)

(2)

Given two graphs *A *= (*V*_*A*_, *E*_*A*_) and *B *= (*V*_*B*_, *E*_*B*_), their neighborhood affinity [12], *NA*(*A*, *B*), is defined as follows to measure the similarity between them,

(3)

For a vertex *v *∈ *V*, the neighborhood graph of *v *consists of *v*, all its neighbors and the edges among them. It is defined as *G*_*v *_= (*V'*, *E'*), where *V' *= {*v*} ∪ {*u*|*u *∈ *V*, (*u*, *v*) ∈ *E*}, and *E' *= {(*u*_*i*_, *u*_*j*_) | (*u*_*i*_, *u*_*j*_) ∈ *E*, *u*_*i*_, *u*_*j *_∈ *V'*}. In *G*_*v*_, there will be some vertices with degree 1 (i.e., only connect with the vertex *v*) and generally the interactions involving these proteins have low reliability with respect to the topological reliability measures in [20-22]. Therefore, all vertices with degree 1 will be removed from *G*_*v*_. Since current PPI data is quite noisy [23], this preprocessing step can help us to filter out possible false positive interactions. The neighborhood graph of *v*, *G*_*v*_, thereafter refers to above remaining graph if it is not empty. As a result, every vertex in *G*_*v *_has at least two neighbors and *Avdeg*(*G*_*v*_) ≥ 2.

### Definition of 'preliminary cores'

A protein-complex core is a small group of proteins which show a high mRNA co-expression patterns and share high degree of functional similarity. It is the key functional unit of the complex and largely determines the cellular role and essentiality of the complex [17,19]. Protein-complex cores and their members often have specific topological properties in PPI networks. For example, a protein in a core often has many interacting partners and protein-complex cores often correspond to small, dense and reliable subgraphs in PPI networks [17]. In addition, complex cores may have overlaps with each other.

According to these properties of protein-complex cores, we first define their possible candidates in the neighborhood graphs, denoted as preliminary cores. A preliminary core in a neighborhood graph *G*_*v *_is a dense subgraph where all its members should show higher significance and have heavier weights [12,13] than those non-members. In particular, we first define a vertex *u *∈ *G*_*v *_as a core vertex if *u*'s degree in *G*_*v *_is larger than or equal to *G*_*v*_'s average degree, i.e., *deg*(*u*) ≥ *Avdeg*(*G*_*v*_). The core graph of *G*_*v *_is defined as the subgraph formed by all the core vertices and their corresponding edges. A preliminary core in *G*_*v *_defined in this paper should satisfy following three constraints: (1) it is a subgraph of the core graph, that is, all its vertices are core vertices, (2) it is dense (with density ≥ *d *and *d *is typically set as 0.7 in [13,14], which is also used in this paper) and (3) it is maximal, that is, none of its supergraphs satisfy the first two constraints.

Above definition of preliminary cores is based on the definition of core vertices, which further relies on the degree distribution of the PPI network. As we know, PPI networks are considered to be scale-free [24]. Therefore, preliminary cores would mostly be formed around the proteins with relatively large degrees. This is reasonable to form preliminary cores because proteins with high degrees in PPI network serve important biological roles [24,25] and tend to be in the "hearts" of protein complexes.

### Protein-complex core mining algorithm

Based on the definition of preliminary cores, we are now ready to describe our proposed algorithm to detect protein-complex cores. In our algorithm, the preliminary cores are first detected from the neighborhood graph of each vertex in the PPI network. Specifically, given a neighborhood graph *G*_*v*_, if its core graph *CG *is dense, *CG *is thus directly predicted as a preliminary core; otherwise, multiple possible preliminary cores would be detected from *CG*. Since some vertices have similar neighborhood graphs, the preliminary cores detected from their neighborhood graphs may have large overlaps, which will result in high redundancy. Hence, a Redundancy-filtering procedure is applied to process preliminary cores and finally generate protein-complex cores by eliminating such kind of redundancy.

Algorithm 1 illustrates the overall framework to detect protein-complex cores. For each vertex *v *in the PPI network *G *= (*V*, *E*), we first construct its neighborhood graph *G*_*v *_and *G*_*v*_'s core graph *CG *in line 3. If *CG *is dense enough, our Core-removal algorithm in line 4 will return it as a preliminary core. If not, Core-removal algorithm may generate several subgraphs of *CG*, which will be further processed to be maximal dense as preliminary core in lines 5–14 (note that a subgraph *sg *obtained from the Core-removal algorithm may not be dense or maximal). If *sg *is not dense, we first iteratively remove vertices with the minimum degree until it is dense in lines 6–9. We may add some core vertices into *sg*, which are highly connected to the vertices in *sg*, to guarantee that *sg *is maximal and dense in lines 10–14. Finally, *sg *will be processed by the Redundancy-filtering procedure in line 15. Note that after filtering the possible redundant preliminary cores, all the remaining preliminary cores in set *SC *will be regarded as protein-complex cores. The details of Core-removal algorithm, as well as Redundancy-filtering procedure, are described in Algorithm 2 and 3 respectively.

Algorithm 1, Protein-complex core mining algorithm

**Input**: The PPI network *G *= (*V*, *E*);

Density threshold *d*;

Neighborhood affinity threshold to filter redundancy *t*.

**Output**: The set of protein-complex cores, *SC*.

1: *SC *= *ϕ*; //initialization

2: **for each **vertex *v *∈ *V ***do**

3:    construct the core graph of *G*_*v*_, *CG *= (*V*_*CG*_, *E*_*CG*_);//*V*_*CG *_= {*u*|*deg*(*u*) ≥ *Avdeg*(*G*_*v*_), *u *∈ *G*_*v*_}

4:    *S *= Core-removal(*CG*);

5:    **for each **element *sg *∈ *S ***do**

6:       **while ***den*(*sg*) <*d ***do**

7:          ; // *deg*(*u*) is *u*'s degree in *sg*

8:          *sg *= *sg *- {*w*};// update *sg *by deleting *w *and its corresponding edges

9:       **end while**

10:       ;// *deg*_1_(*u*) is *u*'s degree in *sg *+ {*u*}

11:       **while ***w *exists and *den*(*sg *+ {*w*}) ≥ *d ***do**

12:          *sg *= *sg *+ {*w*}; // update *sg *by adding *w *and its corresponding edges

13:          ;

14:       **end while**

15:       Redundancy-filtering(*sg*);

16:    **end for**

17: **end for**

#### Core-removal algorithm

**Figure 2:**
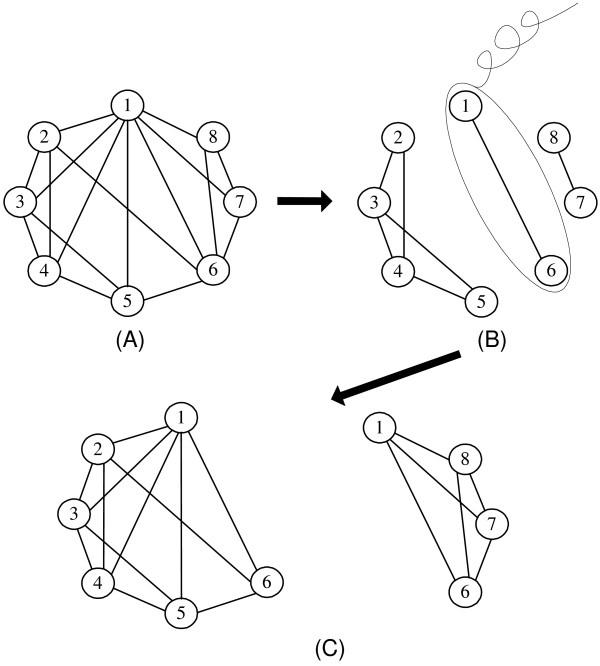
**The diagram of our Core-removal algorithm**. In this example, we assume that (A) shows the core graph of vertex 1's neighborhood graph, denoted as *CG*_1_. The density and average degree of *CG*_1 _is 0.607 and 4.25 respectively. In (B), the core vertices of *CG*_1_, {1, 6}, are removed from *CG*_1 _and two connected components are left in the remaining graph. In (C), {1, 6} are added back into each connected component. Two subgraphs with vertices {1,2,3,4,5,6} and {1,6,7,8} are obtained and finally returned.

Given a vertex *v*, if the core graph of *G*_*v*_, *CG*, is dense enough, core-removal algorithm will return it directly; otherwise, there may be multiple preliminary cores in *CG*. Our core-removal algorithm works as follows. If *CG *is not dense enough, all the core vertices of *CG *are first removed from *CG*, and the remaining graph may consist of a number of connected components. We recursively repeat this procedure to find highly-connected subgraphs in each of above connected components. The removed core vertices are added back into these subgraphs to form larger subgraphs of *CG*, which will be further processed to be maximal dense in Algorithm 1. Algorithm 2 shows the details of our core-removal algorithm and figure 2 also provides an example to illustrate the process of our proposed core-remove algorithm.

**Algorithm 2, Core-removal(*cg*) **// *cg *= (*V*_*cg*_,*E*_*cg*_)

1: *result *= *ϕ*;

2: **if ***den*(*cg*) ≥ *d ***do**

3:    insert *cg *into *result*;

4: **else**

5:    *cv *= {*u*|*u *∈ *V*_*cg*_, *deg*(*u*) ≥ *Avdeg*(*cg*)}; // *deg*(*u*) is *u*'s degree in *cg*

6:    remove all the vertices in *cv *from *cg *and obtain a set of connected components;

7:    **for each **connected component *comp ***do**

8:       *tresult *= Core-removal(*comp*);

9:       **for each **element *tc *∈ *tresult ***do**

10:          insert *tc *∪ *cv *into *result*;

11:       **end for**

12:    **end for**

13: **return ***result*;

#### Redundancy-filtering procedure

Assume that *SC *is the set of all currently detected preliminary cores and *C *= (*V*_*C*_, *E*_*C*_) is a newly detected preliminary core. We will first detect an element *B *= (*V*_*B*_, *E*_*B*_) in *SC*, which has the highest similarity (NA score) with *C*. In Algorithm 3, the procedure Redundancy-filtering(*C*) is used to check and decide whether to discard or preserve the newly detected preliminary core *C*. In particular, if *B *and *C *are not quite similar (with *NA*(*B*, *C*) <*t*), *C *will be inserted into *SC *in lines 2–3; otherwise, we prefer to preserve the preliminary cores that have larger size and density in lines 4–8.

Algorithm 3, Redundancy-filtering(*C*)

1: ;//*B *is *C*'s most similar subgraph in *SC*

2: **if ***NA*(*B*, *C*) <*t ***do**

3:    insert *C *into *SC *(**Inserting**);

4: **else**

5:    **if ***den*(*C*) × |*V*_*C*_| ≥ *den*(*B*) × |*V*_*B*_| **do**

6:       insert *C *into *SC *in place of *B *(**Substituting**);

7: **else**

8:    discard *C *(**Discarding**).

### Protein-complex formation

In previous subsections (the first phase of our COACH method), we have presented our techniques to detect the protein-complex cores. In the second phase, we will extract the peripheral information of each protein-complex core and select reliable attachments cooperating with it to form a protein complex. Given a PPI network *G *= (*V*, *E*), the neighborhood of a complex core *C *= (*V*_*C*_, *E*_*C*_) is defined as *N*(*C*) = {*u*|(*u*, *v*) ∈ *E*, *v *∈ *V*_*C*_, *u *∈ *V*, *u *∉ *V*_*C*_}. *N *(*C*) consists of those direct neighbors of the vertices in the complex core *C*. For a vertex *v *∈ *N*(*C*), *N*_*v *_is the set of all *v*'s neighbors. |*N*_*v *_∩ *V*_*C*_| is the number of vertices in *C *connected with *v*. Thus,  can be used to quantify the closeness between the vertex *v *and the core *C*, denoted as *closeness*(*v*, *C*). Each vertex *v *∈ *N*(*C*) with *closeness*(*v*, *C*) > 0.50 will be selected as an attachment, indicating that selected attachments interact with more than half of the proteins in the core. In this way, the attachments are closely-associated with the complex core, showing that these attachments are in stable and reliable cooperation with the core.

**Figure 3:**
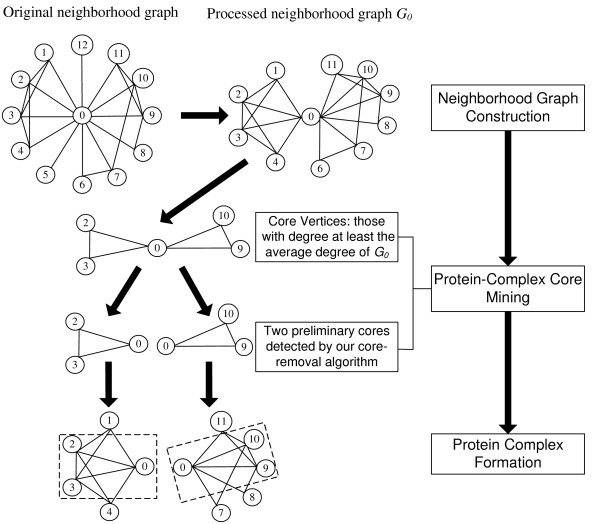
**The diagram of our COACH method**. Our COACH method mainly consists of two stages, protein-complex core detection and complex formation. For simplicity, the Redundancy-filtering procedure is not shown in this figure.

In summary, our COACH method consists of two above stages, protein-complex core detection and complex formation (the available COACH system can be downloaded from . An example in figure 3 illustrates our proposed algorithm to detect protein complexes in PPI networks. For simplicity, the Redundancy-filtering procedure is not shown in this example. As we all know, detecting the complete set of preliminary cores or maximal dense subgraphs is a NP-complete problem (maximal clique finding is a special case when density threshold is 1). However, our heuristic, Core-removal algorithm, detects preliminary cores (not always the complete set of preliminary cores) from the core graphs, which are small-scale subgraphs within each vertex's neighborhood graphs, e.g., the average size of core graphs is 4.30 in DIP data [26] and 5.45 in a denser Krogan et al.'s data [18], respectively. Therefore, our COACH method is very efficient to detect preliminary cores and protein complexes in PPI networks. The Additional File 1 also demonstrates the efficiency and scalability of our COACH method in large-scale random graphs.

## Results and discussions

We have applied our COACH method on two yeast PPI networks. In this section, we will first present in detail the results on DIP data. We perform both comprehensive comparisons among various existing computational methods and validation of our predicted core-attachment structures. The results using Krogan et al.'s data from [18] will also be briefly presented to demonstrate the effectiveness of our proposed technique.

### Comparative evaluation

In this subsection, we compared the performance of our COACH method with other three competing algorithms, DPClus [13], DECAFF [14] and MCL [9,18,27], using DIP data. For comprehensive comparisons, we employed several evaluation measures, including co-annotation, co-localization, functional enrichment of GO terms (p-values), F-measure and coverage rate. For all these methods, the optimal parameters were set to maximize their F-measures. For example, the *inflation *parameter in MCL was set as 1.9 when using DIP data [28]. Note that for fair comparisons, we turned off the filtering step in DECAFF because it used the functional information to filter away possible false positive complexes while other techniques only used topological properties of PPI networks. In addition, a comprehensive comparison between our COACH method and a newly proposed method called CoreMethod [29] is shown in the Additional File 2.

#### F-measure and coverage rate

Table 1 shows the basic information of predictions by various methods. In table 1, MCL predicted 1116 complexes, of which 193 match 242 real complexes; DPClus detected 1143 complexes, of which 193 match 274 real ones and DECAFF detected 2190 complexes, of which 605 match only 243 real ones. Our COACH method managed to predict 746 complexes, out of which 285 match 249 real complexes. In addition, MCODE [12] predicted 182 complexes and correctly matched only 128 real complexes in the benchmark. Both the number of complexes predicted by MCODE and its *N*_*cb *_(*N*_*cb *_is the number of benchmark complexes that are correctly predicted, see Methods section) are far fewer than those of other algorithms. For this reason, MCODE is not included in the later comparisons.

**Table 1:** The results of various algorithms using DIP data

Algorithms	MCODE	MCL	DPClus	DECAFF	COACH
# predicted complexes	182	1116	1143	2190	746
# covered proteins in DIP	1173	4930	2987	1832	1837
*N*_*cp*_	93	193	193	605	285
^*N *^_*cb*_	128	242	274	243	249

**Figure 4:**
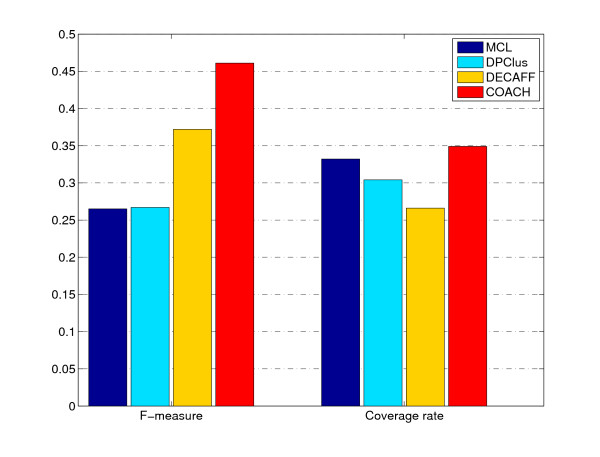
**The performance comparison for various algorithms on DIP data**. This figure shows the F-measure and Coverage Rate of various algorithms on DIP data.

Figure 4 shows the overall comparison in terms of F-measure and coverage rate (see Methods section). On DIP data, the F-measure of COACH is 46.1%, which is 19.6%, 19.4% and 8.9% higher than MCL, DPClus and DECAFF respectively. Our COACH method can achieve the highest F-measure by providing the highest precision and comparable recall, which shows that our method can predict protein complexes very accurately. In figure 4, our COACH method obtains the highest coverage rate of 34.9%, which is 1.7%, 4.5% and 8.3% higher than MCL, DPClus and DECAFF respectively. That is, our predicted complexes can cover the most proteins involved in the real complexes.

**Figure 5:**
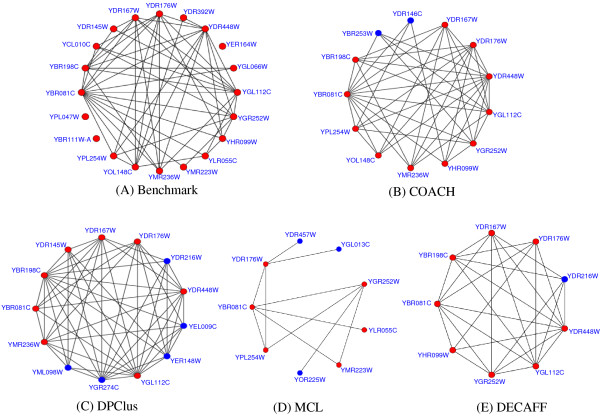
**The SAGA complex predicted by different methods**. In figure 5, (A) shows the real SAGA complex in the benchmark and (B-E) are the SAGA complex predicted by different methods. For each predicted complex, the proteins in red color are involved in the real SAGA complex and those in blue color are not.

Figure 5 illustrates an example, in which our predicted SAGA complex [30] can cover more proteins in the real SAGA complex. In this example, the real SAGA complex in the benchmark consists of 20 proteins (Figure 5A). The complex predicted by our COACH method has 13 proteins and manages to cover 11 proteins (in red color). Meanwhile, MCL, DPClus and DECAFF cover only 6, 8 and 8 proteins of the real SAGA complex respectively.

#### Co-annotation and co-localization

Since protein complexes are formed to perform a specific cellular function, proteins within the same complex tend to share common functions and be co-localized. Generally, higher co-annotation and co-localization scores [27] show that proteins within the same protein complexes tend to share higher functional similarity, and hence they can be used to evaluate the overall quality of predicted protein complexes.

**Figure 6:**
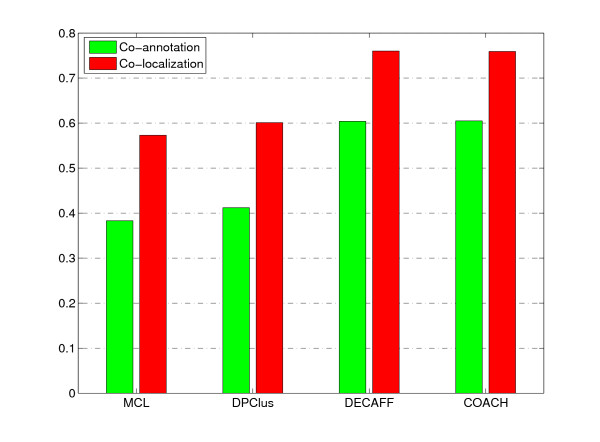
**Co-annotation and co-localization scores of complexes predicted by various methods**. Figure 6 shows the comparison result of various methods in terms of co-annotation and co-localization scores [27].

Figure 6 shows the co-annotation and co-localization scores of complexes predicted by various methods. In terms of these two measures, the complexes predicted by our COACH method are observed to have comparable quality with those predicted by DECAFF, but much better than those predicted by MCL and DPClus.

#### Statistical evaluation of predicted protein complexes

To substantiate the biological significance of our predicted complexes, we calculate their p-values, which represent the probability of co-occurrence of proteins with common functions. Given that proteins in a protein complex are assembled to perform common biological functions, they are thus expected to share common functions. As such, low p-value of a predicted complex generally indicates that the collective occurrence of these proteins in the complex does not happen merely by chance and thus the complex has high statistical significance. In our experiments, the p-values (with Bonferroni correction) of complexes are calculated by the tool, SGD's GO::TermFinder [31].

**Figure 7:**
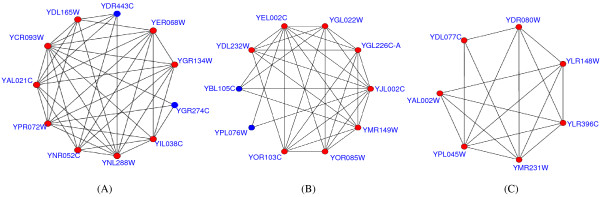
**Examples of protein complexes predicted by COACH method**. In figure 7, the predicted complexes in (A-C) matches CCR4-NOT complex [33], Oligosaccharyl transferase complex (OST complex) [34] and HOPS complex [35], respectively.

Using DIP data, 622 out of 746 complexes predicted by COACH are considered to be significant, with corrected p-value ≤ 0.01 [10]. The proportion of significant complexes over all predicted ones can thus be used to evaluate the overall performance of various methods [32]. Table 2 shows the comparison results based on this measure. In table 2, the majority of our predicted complexes (83.4%) are significant and our COACH method also predicts higher proportion of significant complexes than other three algorithms. Meanwhile, both MCL and DPClus predict many protein complexes with extremely small size (e.g., with two proteins) and generally predicted complexes with small size tend to have large p-values [32] (in table 2, we have discarded the predicted complexes with only one protein when calculating their p-values). Therefore, MCL and DPClus only predicted a small proportion of significant complexes. This result is also consistent with the results in table 1 where both MCL and DPClus achieve very low precision scores. In addition, table 3 shows 10 protein complexes with very low p-values, predicted by our COACH method. The fifth column in table 3 refers to the NA scores between our predicted complexes (in the third column) and real complexes (in the fourth column). The last column shows the number of proteins in the real complexes correctly covered by our predicted complexes. In this table, proteins in bold italic form the protein-complex cores and the rest are attachments. Figure 7 gives three examples of complexes predicted by our COACH method. The first example in figure 7(A) is CCR4-NOT complex [33] (ID = 8). COACH managed to cover 9 out of 12 proteins in the real complex and also had two new proteins (in blue color). The predicted complex in figure 7(B) managed to cover 8 proteins in Oligosaccharyl transferase complex (OST complex) [34] and had two novel proteins (YBL105C and YPL076W) (ID = 9). The third example in figure 7(C) is our predicted HOPS complex, which succeeded to cover all 7 proteins in HOPS [35] (ID = 10). We find that many of our predicted complexes match well with the known complexes. Due to the incompleteness of the benchmark, our non-matched predicted complexes, especially for those with low p-values, may provide potential candidate complexes for biologists to validate.

**Table 2:** Statistical significance of complexes predicted by various methods

Algorithms	MCL	DPClus	DECAFF	COACH
# significant complexes	312	352	1653	622
# predicted complexes	913	1143	2190	746
Proportion (%)	34.2	30.8	75.5	83.4

**Table 3:** Selected protein complexes predicted by our COACH method using DIP data and their p-values

ID	P-values	Predicted protein complexes	Real protein complexes	NA	# common proteins
1	9.85e-33	***YBL084C YDL008W YDR118W YFR036W YGL240W YHR166C YKL022C ***YLR102C ***YLR127C YNL172W YOR249C***	anaphase-promoting complex	0.688	11
2	5.85e-30	YBR154C YDL150W YDR045C YKL144C YKR025W YNL113W YNL151C ***YNR003C YOR116C YOR207C ***YOR224C ***YPR110C ***YPR190C	DNA-directed RNA polymerase III complex	0.765	13
3	7.0e-25	YCR035C YDL111C YDR280W YGR090W ***YGR095C ***YGR158C YGR195W YHR069C ***YHR081W ***YNL189W YNL232W ***YOL021C ***YOL142W YOR001W YOR076C	exosome (RNase complex)	0.805	13
4	5.00e-24	***YBR081C ***YBR198C YBR253W YCL010C YDR167W YDR176W YDR216W ***YDR448W YGL112C YGR252W ***YHR099W YMR236W YPL254W	SAGA complex	0.452	11
5	9.90e-23	***YBR081C ***YBR198C YDR167W ***YDR176W ***YDR392W ***YDR448W ***YEL009C ***YER148W ***YGL112C YGR274C YHR099W YMR236W ***YOL148C ***YPL254W YPR086W	SLIK (SAGA-like) complex	0.475	11
6	6.61e-23	YBL093C YBR193C ***YBR253W ***YCR081W YDL005C YDL140C YDR308C YER022W YGR104C YHR041C YHR058C ***YLR071C YNL236W YOL051W YOL135C ***YOR174W YPR070W	RNA polymerase II mediator complex	0.602	16
7	1.57e-20	***YAR003W YBR175W YBR258C YDR469W YHR119W ***YKL018W ***YLR015W YPL138C***	COMPASS complex	1.0	8
8	1.36e-19	***YAL021C YCR093W YDL165W YDR443C YER068W YGR134W YGR274C YIL038C YNL288W YNR052C YPR072W***	CCR4-NOT complex	0.614	9
9	1.43e-15	YBL105C YDL232W ***YEL002C YGL022W ***YGL226C-A ***YJL002C ***YMR149W YOR085W YOR103C YPL076W	OST complex	0.71	8
10	1.66e-13	YAL002W YDL077C ***YDR080W YLR148W YLR396C YMR231W YPL045W***	HOPS complex	1.0	7

### Validation of core-attachment structures

We first analyzed the difference between the cores as consistent functional "hearts" and attachments as "secondary" units by using various biological evidences, such as the GO annotations and gene expression data. Then, we validated a few examples of predicted protein complexes using biological knowledge from literature.

#### Analysis of protein-complex cores

Proteins within the same complex core should have higher degree of functional similarities and tend to co-localize to the same subcellular compartment than those attachments [17,19]. Two interacting proteins (or an interaction) can have a similarity score based on their GO terms or gene expression profiles. In our experiments, functional similarity between two proteins is calculated based on the method in [36] and expressional correlation is measured by the Pearson correlation coefficient. The overall quality of all interactions involved in protein-complex cores is an aid to analyze those complex cores. Using DIP data, we managed to identify 746 complex cores, involving 3886 interactions among 1536 proteins. Table 4 shows the average similarity of all these interactions, using two sub-ontologies of GO (BP-"Biological Process" and CC-"Cellular Component") and gene expression data respectively. We also obtained the average similarity of two other sets of interactions, all the interactions in DIP data and those involved in our inferred protein complexes. In table 4, we can find that interactions within protein complexes have higher similarities than those in the whole DIP PPI data, while interactions within complex cores even have higher similarity than those in complexes, which indicates the cores' biological meanings.

**Table 4:** Average similarity of interactions involved in protein-complex cores, protein complexes and DIP data, respectively

Interactions	Biological Process	Cellular Component	Gene Expression
In COACH complex cores	0.558	0.706	0.274
In COACH complexes	0.502	0.674	0.264
In DIP data	0.357	0.570	0.235

**Figure 8:**
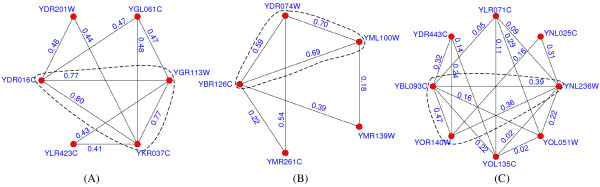
**Predicted complexes with interactions scored by gene-expression correlations**. In figure 8, the proteins in the dashed circles form the cores of those predicted complexes and each interaction is attached with the gene-expression correlation between its two interacting proteins. The predicted complexes in (A-C) match the DASH complex [37], trehalose-6-phosphate synthase/phosphatase complex [38] and RNA polymerase II mediator complex [39], respectively.

**Figure 9:**
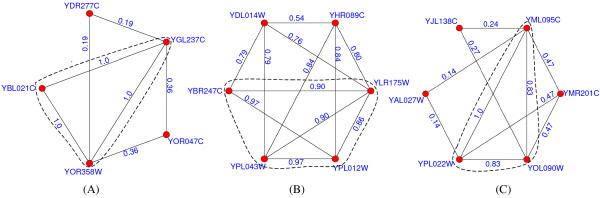
**Predicted complexes with interactions scored by functional similarities (using BP)**. In figure 9, each interaction is attached with the functional similarity (using BP) between its two interacting partners. The predicted complexes in (A-C) match the CCAAT-binding factor complex [40], Cbf5-Nop10 complex [41] and nucleotide-excision repair factor 1 complex [42], respectively.

**Figure 10:**
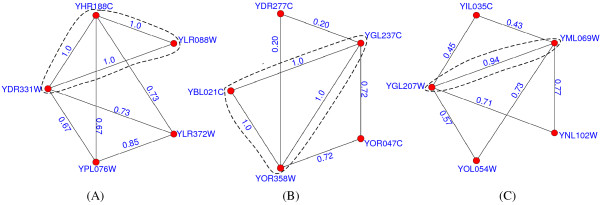
**Predicted complexes with interactions scored by functional similarities (using CC)**. In figure 10, each interaction is attached with the functional similarity (using CC) between its two interacting partners. The predicted complexes in (A-C) match the GPI-anchor transamidase complex [43], CCAAT-binding factor complex and FACT complex [44], respectively.

Figures 8, 9, 10 present some examples in which proteins within the protein-complex cores share much higher gene-expression correlations or functional similarities. Proteins in the dashed circles form the cores of those predicted complexes in these figures.

In figure 8, each interaction within the predicted complexes is annotated with the gene-expression correlation between its two interacting proteins. The predicted complex in (A) consists of 6 proteins and matches the DASH complex [37]. In our predicted DASH complex, proteins YDR016C, YGR113W and YKR037C form the complex core. All 3 interactions in the core have a average gene-expression correlation of 0.78, which is much higher than that of interactions not in the core (0.45). The predicted complexes in (B) and (C) match the trehalose-6-phosphate synthase/phosphatase complex [38] and RNA polymerase II mediator complex [39], respectively. The average gene-expression correlations for interactions within and without the cores are 0.66 and 0.33 in (B) and 0.41 and 0.17 in (C), respectively.

In figure 9, each interaction is annotated with the similarity of biological processes between its two interacting partners. The predicted complex in (A) matches the CCAAT-binding factor complex [40]. In this predicted complex, the core consists of proteins YBL021C, YGL237C and YOR358W, which exactly share the same GO annotations, e.g., all of them are involved in both the transcription (GO:0006350) and regulation of carbohydrate metabolic process (GO:0006109). Two predicted attachments, proteins YDR277C and YOR047C, are involved in glucose transport (GO:0015758) and glucose metabolic process (GO:0006006), respectively. Obviously, the interactions not in the core have a much lower average functional similarity (0.28). The predicted complexes in (B) and (C) match Cbf5-Nop10 complex [41] and nucleotide-excision repair factor 1 complex [42], respectively. The average similarities of biological processes for interactions within and without the cores are 0.93 and 0.77 in (B) and 0.89 and 0.31 in (C), respectively. Similarly in figure 10, each interaction is annotated with the similarity of cellular components between its two interacting partners. The predicted complexes in (A-C) match the GPI-anchor transamidase complex [43], CCAAT-binding factor complex (note that the predicted complex in (B) is the same one in figure 9(A)) and FACT complex [44], respectively. The average similarities of cellular components for interactions within and without the cores are 1.0 and 0.73 in (A), 1.0 and 0.46 in (B) and 0.94 and 0.61 in (C), respectively.

From table 4 and figures 8, 9, 10, it is clearly observed that interactions within or not in the protein-complex cores have different-level similarity scores. This fact supports that proteins in complexes should be categorized into different organization levels, i.e., core member level and attachment level. All above evidences also constitute proofs that our identified protein-complex cores are mostly biological hearts of protein complexes. Meanwhile, additional remarks in the Appendix show that interactions within the cores tend to be reliable, signifying another evidence for the importance of cores.

#### Validating examples for core-attachment structures

To illustrate the organization of our predicted complexes, we further analyzed the second and third predictions in figure 7.

In the OST complex as shown in figure 7(B), the core consists of 3 proteins (YEL002C, YGL022W and YJL002C). This is also reported in Gavin et al.'s analysis [17]. In addition, our method correctly identified 5 known attachments (YDL232W, YGL226C-A, YMR149W, YOR085W and YOR103C) and predicted two new attachments (YBL105C and YPL076W). Since the protein YML019W in the real OST complex interacts with only one protein (YJL002C) within the core in DIP data, our predicted complex did not identify it as an attachment. However, the interactions between YML019W and all three proteins in the core have been reported in [3]. As more PPI data accumulated, we can expect our COACH can work even better.

As to the HOPS complex in figure 7(C), proteins YDR080W and YDL077C are its attachments and the core consists of proteins YLR148W, YLR396C, YMR231W, YPL045W and YAL002W. Biological experiments show the core have the function of vacuole protein sorting [45]. With the help of attachments YDR080W and YDL077C, this complex can perform the function of homotypic vacuole fusion [35]. This demonstrates that our computational discovery is consistent with the current biological knowledge, indicating that some novel knowledge could be discovered by our proposed method. Of course, biological experiments are necessary for further validating.

### Effect of the parameter *t*

**Figure 11:**
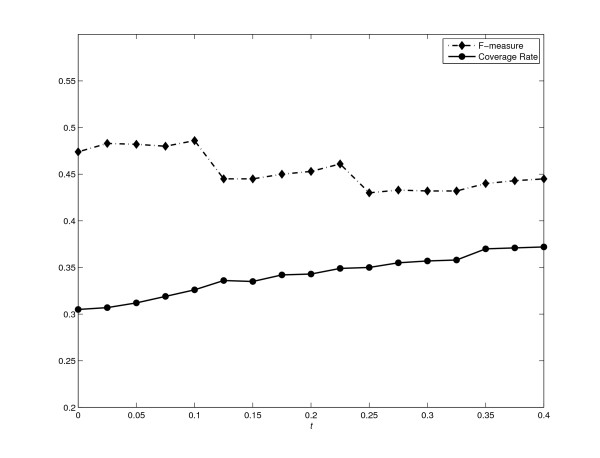
**The effect of *t***. Figure 11 shows how the variation of parameter *t *affect the F-measure and Coverage Rate of our COACH method.

Recall that COACH method employs a user-defined parameter *t *(see Algorithm 3) to filter redundant preliminary cores. It is obvious that overlaps among protein-complex cores are allowed when *t *> 0 and are not allowed when *t *= 0. We now investigate how the variation of *t *affects the performance of our COACH method. Figure 11 shows the F-measure and coverage rate of our COACH method under different values of *t*, using DIP data.

Firstly, the number of predicted complexes increases with the increase of *t*, i.e., COACH generates 268, 715 and 1040 complexes under *t *= 0, 0.2 and 0.4 respectively. This is because the bigger the value of *t*, the more overlaps among protein-complex cores are allowed, resulting in more predicted complexes. With more complexes predicted, it is reasonable that more proteins in the benchmark complexes are covered with increasing the value of *t*. However, the number of cases that multiple predicted complexes match the same real complex (also denoted as the redundancy in predicted complexes) is also increased with the increase of *t*. For example, 267 correct predictions match 247 real complexes when *t *= 0.2 (1.08 predicted complexes match one real complex on average), while 371 match only 253 when *t *= 0.4 (1.47 predicted complexes match one real complex on average).

Secondly, as we increase the values of *t*, the curve of the resulting F-measure in figure 11 is observed to have 3 distinct and stable ranges for values of *t*, i.e., [0, 0.1], [0.125, 0.225] and [0.25, 0.4] respectively. Higher F-measure is achieved in the first range (*t *∈ [0, 0.1]), which sacrifices the Coverage Rate, resulting the lowest Coverage Rate compared with those in other two ranges. Additionally in this range, COACH only generates a small number of complexes (e.g., it predicts 308 complexes, 145 out of which match 211 real complexes when *t *= 0.05). On the contrary, higher Coverage Rate are achieved in the third range (*t *∈ [0.25, 0.4]), which sacrifices the F-measure. However, as stated above, the redundancy in our predicted complexes would become more severe with the increase of *t*. To reduce the redundancy involved in predicted complexes, as well as to encourage one-to-one matching between predicted complexes and real ones, we recommend that the suitable setting of *t *would be in the second range i.e., *t *∈ [0.125, 0.225]. In fact, the performance of COACH does not change significantly in this range. For the setting of *t *= 0.225 in our experiments, COACH can achieve a good balance of both F-measure and Coverage Rate.

### Results using Krogan et al.'s PPI data

**Figure 12:**
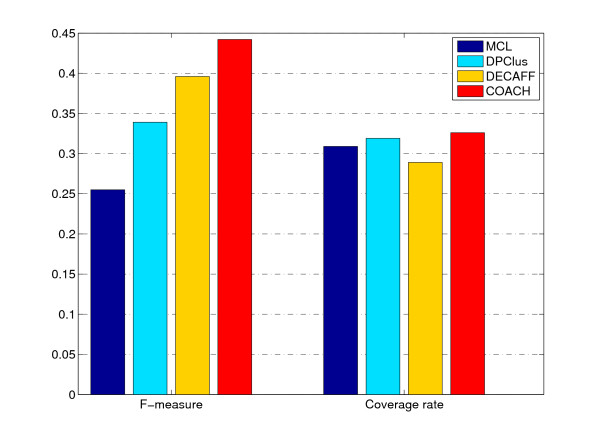
**F-measure and Coverage Rate of various methods for Krogan et al.'s data**. The F-measure and Coverage Rate of each method using Krogan et al.'s data (*t *= 0.225) are shown in figure 12.

We also performed our COACH on Krogan et al.'s PPI data (see Methods section). The F-measure and coverage rate of each method using this data (*t *= 0.225) are shown in figure 12. The F-measure of our COACH is 44.2%, which is 18.7%, 10.3% and 4.6% higher than MCL, DPClus and DECAFF respectively. From the perspective of Coverage Rate, our COACH still performs the best as shown in figure 12.

In particular, we check both *N*_*cp *_and *N*_*cb *_(see Method section) of DECAFF and find that DECAFF generates protein complexes with some redundancy on both DIP and Krogan et al.'s PPI data. In other words, the complexes predicted by DECAFF overlap a lot with each other. For example, DECAFF predicts 2190 complexes using DIP data, 605 of which match 243 real complexes. Even, it predicts 2143 complexes using Krogan et al.'s data, 759 of which only match 192 real complexes (almost 4 correct predictions match a real one on average). The ratio *N*_*cp*_/*N*_*cb *_of DECAFF is much higher than that of other methods, which suggests that some post-processing (such as, clustering highly overlapping complexes) is needed for DECAFF.

## Conclusion

Protein complexes are key molecular entities to perform cellular functions. The increasing amount of protein-protein interaction (PPI) data has enabled us to detect protein complexes from PPI networks. However, current computational methods only focus on detecting dense subgraphs in PPI networks as protein complexes but ignore their inherent organization. Hence, new approaches that can provide insights into the organization of protein complexes are greatly desired.

In this paper, we proposed a core-attachment based method to detect protein complexes from PPI networks. We first mined the protein-complex cores from the neighborhood graphs and then formed protein complexes by including attachments into cores. The evaluation and analysis of our predictions demonstrated the following advantages of our COACH method over the state-of-the-art techniques. First, Our proposed method is fundamentally different from existing methods. It provides insights into the inherent organization of protein complexes while existing methods mainly focus on detecting dense graphs. Proteins within the same protein-complex core detected by our method have high functional similarity and tend to be co-localized, indicating that protein cores are possible biological hearts of protein complexes. Second, COACH has achieved significantly higher F-measure and Coverage rate than existing methods. Thus, our predicted complexes match very well with benchmark complexes. In addition, COACH also performs very well in terms of other measures such as co-annotation, co-localization and p-values, indicating that COACH can predict protein complexes very accurately. Our identified complexes, therefore, could be probably the true complexes to help the biologists to get novel biological insights. As we know, a protein complex is often formed by multiple proteins which have permanent and stable relations with each other. However, current PPI networks do not differentiate the types of interactions. Recent studies demonstrate that proteins structures and binding interfaces [46,47] are of help to address the above issue. One of our future studies is to integrate current PPI data and available structural information to detect protein complexes with core-attachment structures.

## Methods

### Experimental data

In our experiments, we performed our COACH method on two yeast PPI networks. One is from DIP (the Database of Interacting Proteins [26]), which consists of 17203 interactions among 4930 proteins. Krogan et al.'s PPI data [18] consists of 14077 reliable interactions involving 3581 proteins (with a cut-off of 0.101 as shown in their supplementary table S8). For evaluating our identified complexes, the set of real complexes from [27] was selected as benchmark. This benchmark set consists of 428 protein complexes, from three sources: (I) MIPS [48], (II) Aloy et al. [49] and (III) SGD database [50] based on Gene Ontology (GO) annotations. In addition, the gene expression data was downloaded from Eisen's lab [51] and GO data was downloaded from [52].

### F-measure

The neighborhood affinity score (in equation 3) between a predicted complex *p *and a real complex *b *in the benchmark, *NA*(*p*, *b*), is used to determine whether they match with each other. If *NA*(*p*, *b*) ≥ *ω*, they are considered to be matching (*ω *is set as 0.20 in [12,14], which is also used in this paper). We assume that *P *and *B *are the sets of complexes predicted by a computational method and real ones in the benchmark, respectively. *N*_*cp *_is the number of correct predictions which match at least a real complex and *N*_*cb *_is the number of real complexes that match at least a predicted one. Precision and recall are defined as follows [15]:

(4)

F-measure, as the harmonic mean of precision and recall, can be used to evaluate the overall performance of the different techniques [8,14].

### Coverage rate

Coverage rate [27,53] is applied to show how many proteins in the real complexes can be covered by the predicted complexes. Given *n *benchmark complexes and *m *predicted complexes, *T*_*ij *_is the number of proteins in common between *i*^*th *^benchmark complex and *j*^*th *^predicted complex. Coverage rate is then defined as:

(5)

where *N*_*i *_is the number of proteins in the *i*^*th *^benchmark complex.

## Authors' contributions

MW and XL conceptualized and designed the method and drafted the manuscript together. MW was responsible for the implementation. CKK and SKN participated in discussion and conceptualization as well as revising the draft. All authors read and approved the manuscript.

## Appendix

**Remark 1**. A preliminary core detected from vertex *v*'s neighborhood graph *G*_*v *_will definitely contain the vertex *v*.

Since vertex *v *links to all other vertices in *G*_*v *_and has the maximum degree, it is easy to understand the remark 1.

**Remark 2**. Each interaction in a complex core tend to be reliable.

Let a complex core *pc *= (*V*_*pc*_, *E*_*pc*_) be a preliminary core detected from *G*_*v*_. We discuss the reliability of interactions within this complex core, based on following 2 cases.

Case 1: *Avdeg*(*G*_*v*_) = 2. In this situation, *G*_*v *_is protein-triangle and also a preliminary core itself, i.e., *pc *= *G*_*v*_. Moreover, each protein pair in *G*_*v *_has a common neighbor (an alternative path through one prortein) and protein interactions within this topology tend to be reliable [21].

Case 2: *Avdeg*(*G*_*v*_) > 2. For every interaction *e *= (*u*_1_,*u*_2_) ∈ *E*_*pc*_, *e *will be in one of two following cases.

**Case 2.1, ***u*_1 _= *v*, *u*_2 _≠ *v*. Since *deg*(*u*_2_) ≥ *Avdeg*(*G*_*v*_) > 2, *u*_2 _will have at least another 2 neighbors besides *u*_1_, which are common neighbors between *u*_1 _and *u*_2_. In this case, *e *is demonstrated to have higher reliability than those in Case 1[22,54]. **Case 2.2, ***u*_1 _≠ *v*, *u*_2 _≠ *v*. *u*_1 _and *u*_2 _will have at least a common neighbor, namely *v*. *e *= (*u*_1_,*u*_2_) thus has a reliable alternative path {*u*_1 _- *v *- *u*_2_}. *e *is also shown to be with high reliability in [55].

## Supplementary Material

Additional file 1 **The running time of our COACH method over random graphs**. Additional file 1 shows the running time of our COACH method on two kinds of random graphs and demonstrates that COACH is efficient in large-scale graphs. Click here for file

Additional file 2 **The comparison between our COACH method and the CoreMethod**. Additional file 2 first briefly introduces the CoreMethod. A comprehensive comparison between our COACH method and the CoreMethod is then presented in this file. Click here for file
