# Supplementary material for: A core-attachment based method to detect protein complexes in PPI networks
Source: BMC Bioinformatics. 2009 Jun 2;10:169. doi: 10.1186/1471-2105-10-169 (PMC2701950; doi:10.1186/1471-2105-10-169)
Supplement: Additional file 1 — The running time of our COACH method over random graphs. Additional file 1 shows the running time of our COACH method on two kinds of random graphs and demonstrates that COACH is efficient in large-scale graphs. [file 1471-2105-10-169-S1.pdf]

A Core-Attachment based Method to Detect Protein Complexes in PPI Networks

Min Wu[1], Xiaoli Li[2], Chee-Keong Kwoh[1], See-Kiong Ng[2]
{wumi0002,asckkwoh}@ntu.edu.sg and {xlli,skng}@i2r.a-star.edu.sg

[1] School of Computer Engineering, Nanyang Technological University, Singapore
[2] Institute for Infocomm Research, 1 Fusionopolis Way, Singapore.

Additional File 1

Figure 1 shows the running time of our COACH method over two kinds of random graphs (*i.e.*, Power-law random graphs and Geometric random graphs) on the workstation with 3.4GHz Dual Core processors and 3GB RAM. In power-law graphs, the degree distribution follows a power-law, *i.e.*, $P(k) \sim k^{-\alpha}$, where $P(k)$ is the probability of a nodes with a degree of k and $\alpha > 0$. Geometric random graphs are constructed by dropping n points randomly into the unit square and adding edges between two nodes with distance less than a pre-defined threshold.

In our experiments, after a power-law graph is randomized, a geometric random graph will be generated with the same number of nodes and edges as the power-law graph. Given a fixed number of nodes, we generated 50 pairs of power-law graphs and geometric random graphs and then calculated the average running time of COACH over them. Figure 1 thus demonstrates that our COACH method is efficient in large-scale graphs.

The basic information of these random graphs, *i.e.*, the average number of edges, is shown in figure 2. We also note that the average degrees ($2 \times \#Edges/\#Nodes$) of the random graphs slightly increase as the number of nodes increases.

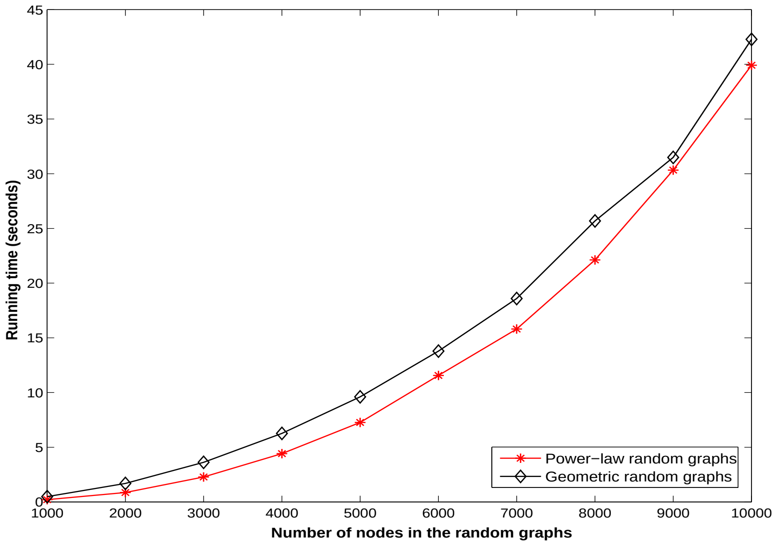

Fig. 1. The running time of our COACH on the random graphs.

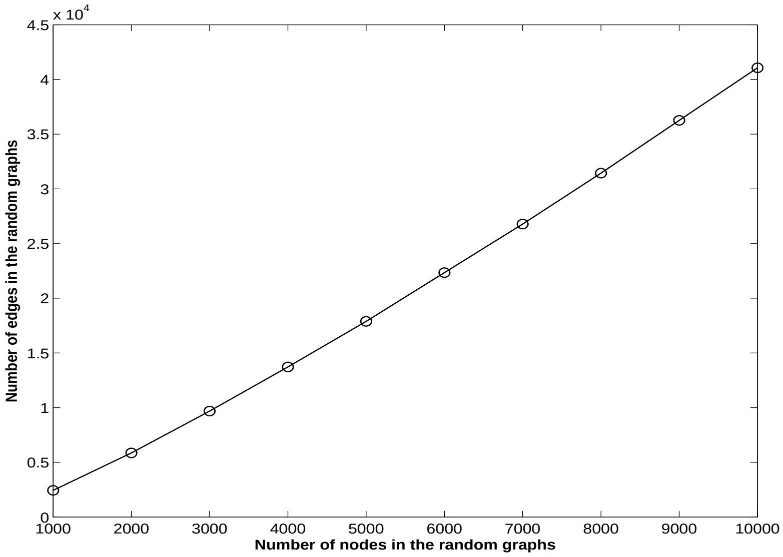

Fig. 2. The average number of edges in the random graphs in our experiments.
