# Supplementary material for: A core-attachment based method to detect protein complexes in PPI networks
Source: BMC Bioinformatics. 2009 Jun 2;10:169. doi: 10.1186/1471-2105-10-169 (PMC2701950; doi:10.1186/1471-2105-10-169)
Supplement: Additional file 2 — The comparison between our COACH method and the CoreMethod. Additional file 2 first briefly introduces the CoreMethod. A comprehensive comparison between our COACH method and the CoreMethod is then presented in this file. [file 1471-2105-10-169-S2.pdf]

A Core-Attachment based Method to Detect Protein Complexes in PPI Networks

Min Wu[1], Xiaoli Li[2], Chee-Keong Kwoh[1], See-Kiong Ng[2]
{wumi0002,asckkwoh}@ntu.edu.sg and {xlli,skng}@i2r.a-star.edu.sg

[1] School of Computer Engineering, Nanyang Technological University, Singapore
[2] Institute for Infocomm Research, 1 Fusionopolis Way, Singapore.

Additional File 2

Recently, Leung et al. also developed an approach called CoreMethod to detect protein complexes in PPI networks by identifying their cores and attachments separately [2]. Next, we briefly introduce the CoreMethod and then compare it with our COACH method.

In the CoreMethod, the probability for two proteins to be in the same protein-complex core (denoted as the p-value) is mainly determined by two factors: whether these two proteins interact or not and the number of the common neighbors between them. The CoreMethod then calculates the p-values for all pairs of proteins (i.e., pair-wise fashion) to identify protein-complex cores. However in our COACH method, preliminary cores are first detected by locally searching highly interactive proteins in each vertex's neighborhood graph. Protein-complex cores are then obtained by filtering out redundant preliminary cores (please see the Algorithm section). As such, both approaches for detecting protein-complex cores are quite different in computational essence. To detect cores, our COACH performs local search within vertex's neighborhood graphs while the CoreMethod computes the p-values between all the proteins in the whole PPI networks. Therefore, COACH is able to achieve significant computational speedup against the CoreMethod.

In addition, we notice that there is another difference between the two methods. Protein-complex cores detected by the CoreMethod are non-overlapping whereas our COACH method is able to detect the overlapping cores. Biologically, certain proteins can serve as coordinators and thus can involve in multiple cores. In fact in Gavin et al.'s nature paper [1], it has shown that the different complex cores have some overlaps, demonstrating that our COACH method has matched well with the biological evidences than the CoreMethod.

Interestingly, both the CoreMethod and COACH select attachments to form protein complexes, based on the same strategy. That is, a protein will be included as an attachment to a core if more than half of proteins within the core are its interacting partners. Furthermore, we employed various evaluation measures, such as F-measure and Coverage rate (please see Methods section), to compare the overall quality of complexes predicted by these two methods. Table 1 shows the comparison results between the CoreMethod and our COACH method, in terms of precision, recall, F-measure and Coverage rate. On DIP data, the precision of COACH is 38.2%, which is almost as 3 times as that of the

CoreMethod. The recall of the CoreMethod (59.8%) is slightly better than that of COACH (58.2%). As such, the resulting F-measure of COACH is significantly (25%) higher than that of the CoreMethod. Similarly on Krogan et al.'s data, the precision and F-measure are significantly better than those of the CoreMethod (the overall F-measure of COACH is 19.2% higher than CoreMethod), while the recall of the CoreMethod is higher than that of COACH. The Coverage rates of the two methods are comparable on these two PPI networks - CoreMethod is slightly (around 0.7%) higher than that of COACH method. However, the overall performance of COACH is much better than CoreMethod.

Table 1. Comparative analysis between CoreMethod and COACH.

Performance	DIP data		Krogan et al.'s data	
	CoreMethod	COACH	CoreMethod	COACH
Precision	0.128	0.382	0.163	0.432
Recall	0.598	0.582	0.535	0.453
F-measure	0.211	0.461	0.250	0.442
Coverage Rate	0.356	0.349	0.334	0.328

References

1. A.C. Gavin, P. Aloy, P. Grandi, R. Krause, M. Boesche, M. Marzioch, C. Rau, L. J. Jensen, S. Bastuck, B. Dumpelfeld, and *et al.* Proteome survey reveals modularity of the yeast cell machinery. *Nature*, 440(7084):631–636, 2006.
2. H. Leung, Q. Xiang, S. Yiu, and F. Chin. Predicting protein complexes from ppi data: A core-attachment approach. *Journal of Computational Biology*, 16(2):133–144, 2009.
